# Acrodermatitis due to zinc deficiency after combined vertical gastroplasty with jejunoileal bypass: case report

**DOI:** 10.1590/S1516-31802012000500010

**Published:** 2012-11-13

**Authors:** Selma Freire de Carvalho Cunha, Gilson Antônio Pereira Gonçalves, Julio Sérgio Marchini, Ana Maria Ferreira Roselino

**Affiliations:** I MD, PhD. Assistant Professor, Division of Nutrology, Faculdade de Medicina de Ribeirão Preto (FMRP), Universidade de São Paulo (USP), Ribeirão Preto, São Paulo, Brazil.; II MD. Resident in Dermatology, Hospital das Clínicas, Faculdade de Medicina de Ribeirão Preto (FMRP), Universidade de São Paulo (USP), Ribeirão Preto, São Paulo, Brazil.; III PhD. Full Professor, Division of Nutrology, Department of Internal Medicine, Faculdade de Medicina de Ribeirão Preto (FMRP), Universidade de São Paulo (USP), Ribeirão Preto, São Paulo, Brazil.; IV MD, PhD. Associated Professor, Division of Dermatology, Department of Internal Medicine, Faculdade de Medicina de Ribeirão Preto (FMRP), Universidade de São Paulo (USP), Ribeirão Preto, São Paulo, Brazil.

**Keywords:** Acrodermatitis, Zinc, Bariatric surgery, Protein deficiency, Jejunoileal bypass, Gastroplasty, Acrodermatite, Zinco, Cirurgia bariátrica, Deficiência de proteína, Derivação jejuno-ileal, Gastroplastia

## Abstract

**CONTEXT:**

Nutritional complications may occur after bariatric surgery, due to restriction of food intake and impaired digestion or absorption of nutrients.

**CASE REPORT:**

After undergoing vertical gastroplasty and jejunoileal bypass, a female patient presented marked weight loss and protein deficiency. Seven months after the bariatric surgery, she presented dermatological features compatible with acrodermatitis enteropathica, as seen from the plasma zinc levels, which were below the reference values (34.4 mg%). The skin lesions improved significantly after 1,000 mg/day of zinc sulfate supplementation for one week.

**CONCLUSIONS:**

The patient’s evolution shows that the multidisciplinary team involved in surgical treatment of obesity should take nutritional deficiencies into consideration in the differential diagnosis of skin diseases, in order to institute early treatment.

## RESUMO

**CONTEXTO:**

Complicações nutricionais podem ocorrer após cirurgia bariátrica, pela restrição no consumo de alimentos e por comprometimento do processo digestivo e absortivo.

**RELATO DO CASO:**

Após ter sido submetida a gastroplastia vertical e derivação jejuno-ileal, uma paciente apresentou acentuada perda de peso e desnutrição proteica. Sete meses após a cirurgia bariátrica, manifestou-se quadro dermatológico compatível com acrodermatite enteropática, verificado a partir dos níveis plasmáticos de zinco (34,4 mg%), que se situavam abaixo dos valores de referência. As lesões cutâneas melhoraram significativamente após 1.000 mg/dia de suplementação de sulfato de zinco, por uma semana.

**CONCLUSÕES:**

A evolução da paciente mostra que a equipe multiprofissional envolvida no tratamento cirúrgico da obesidade deve considerar as deficiências nutricionais no diagnóstico diferencial das doenças cutâneas, a fim de instituir precocemente o tratamento.

## INTRODUCTION

Bariatric surgery provides better control over comorbidities such as hypertension, diabetes, hyperlipidemia and obstructive sleep apnea, thereby leading to improved quality of life for morbidly obese patients.^1^ However, nutritional deficiencies are common during the postoperative period following bariatric surgery,^2^ because of anatomical alterations in the gastrointestinal tract.^3^ There are a number of different bariatric procedures available, and, in general, patients who undergo malabsorptive procedures are at higher risk of long-term nutrition-related complications than are those undergoing restrictive procedures.^2^^,^^4^ Vitamins B_12_ and D, iron and calcium are the most common deficiencies, whereas cases of greater severity are a consequence of thiamine, folate and liposoluble vitamin deficiencies,^3^^,^^5^ despite routine supplementation.^6^ Regarding zinc, many studies have documented reduced levels of this mineral following bariatric surgery,^7^^,^^8^ although there are few reports on clinical manifestations subsequent to the surgical procedure.^9^

Acrodermatitis enteropathica is a relatively rare autosomal recessive metabolic disorder affecting zinc absorption.^10^^,^^11^ Acquired zinc deficiency may be secondary to low mineral supply stemming from deficient intake, nutrient-drug interaction or diminished uptake due to dietary factors^12^ such as phytate.^13^ Alcoholism, malabsorption, chronic renal diseases, chronic debilitating disorders and extensive burns can also trigger acquired zinc deficiency.^14^

Zinc was recognized as essential for humans in the early 1960s. Since then, it has been demonstrated that this metal is a component of over 300 enzymes. Zinc deficiency has also been reported to cause growth retardation and delayed puberty in adolescents, hypogonadism in males, cognitive impairment, mental lethargy, taste abnormalities, diarrhea, poor appetite, weight loss, immune dysfunction, alopecia, delayed wound healing and bullous-pustular dermatitis.^14^

There are various hypotheses regarding the pathogenesis of zinc deficiency in cutaneous lesions. Nutritional deficiencies manifest themselves in cells with high turnover rates, such as keratinocytes. Skin signs of zinc deficiency include erythematous desquamative patterns and eczematous plaques, which may eventually evolve into vesiculobullous lesions in the periorificial, skin fold and acral areas. Nail abnormalities such as onychodystrophy and paronychia, as well as ocular findings and mucosal lesions such as stomatitis, angular cheilitis, blepharitis, conjunctivitis and photophobia, are less common. Histopathological examination is not specific, and the most usual findings are parakeratosis, papillary dermal edema with massive ballooning and slightly pale keratinocytes; intra-epidermal bullae may also be detected.

The case reported here is of a female patient who presented with cutaneous lesions attributed to zinc deficiency, which was detected seven months after vertical gastroplasty and jejunoileal bypass, a non-routine combination of techniques in bariatric surgery. The clinical manifestations of zinc deficiency, laboratory data and therapeutic response following zinc supplementation are discussed. 

## CASE REPORT

In October 2008, a 30-year-old female patient was admitted to the nutrology services of a university hospital complaining of apathy, irritability, pronounced reduction in walking ability and cutaneous lesions. Seven months prior to admittance, she had undergone laparoscopic bariatric surgery. At the time of the surgical procedure, she had a body mass index (BMI) of 44.5 kg/m^2^. For the bariatric surgery, two surgical techniques were combined, namely sleeve gastrectomy or vertical banded gastroplasty and jejunoileal bypass, as described by Souza.^15^ The latter is considered to be a modification of the Payne surgical procedure and involves reversible side-to-side anastomosis between the jejunum and ileum and placement of a silicone ring in the jejunum, in a segment located after the bypass.

During the first six postoperative months, the patient presented several complications, such as enterocutaneous fistula, surgical wound infection, subphrenic abscess and pulmonary thromboembolism. Even after resolution of the postsurgical complications, she still suffered from hyporexia, nausea and vomiting, which implied large restriction of food intake and weight loss of 58 kg (49% of the patient’s preoperative weight). 

About seven months postoperatively, she experienced cutaneous lesions characterized by erythema, fine desquamation, xerosis and diffuse pruritus, which manifested after exposure to sunlight. She was initially treated with oral anti-allergic drugs, without any improvement. According to the patient’s account, a cutaneous biopsy prior to hospital admission had shown an “allergic process and vitamin deficiency”. On that occasion, vitamin B_12_ and thiamine supplementation were started, with relative improvement in the patient’s general health status and appetite, but no changes in the cutaneous picture.

Although the patient had had diarrhea during the postoperative months, she was evacuating twice a day (pasty or semiliquid stool) at the time of admittance to our service. A 24-hour diet questionnaire was applied, and nutrient intake analysis was conducted by means of specific software (NutWin Profissional^®^ 1.5 software, Universidade Federal de São Paulo [Unifesp], São Paulo, Brazil). Low daily calorie intake (1,164 kcal) and adequate proteins, vitamins, and mineral ingestion were documented. Zinc consumption (8.8 mg/day) on the days prior to evaluation was in accordance with the estimated average requirement (EAR) for this mineral (6.8 mg/day for women).^16^ The patient was using multivitamin preparations that did not contain minerals, and antidepressants without any potential pharmacological interaction with zinc absorption, metabolism or excretion. 

**Figure 1.**
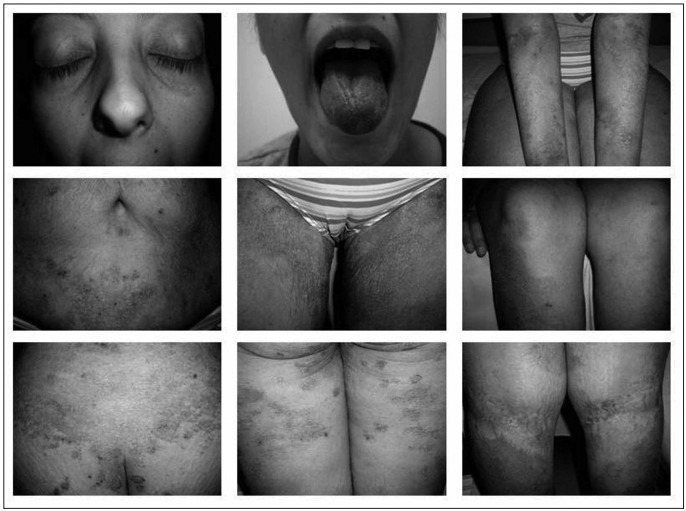
Female patient with acrodermatitis enteropathica following bariatric surgery: The patient presented periorbital hyperchromia, bright red lingual erythema and papillary hypotrophy on the edges of the tongue. Lenticular and erythematous hyperchromic plaques skin lesions, fine scales and hematic crusts, associated with xerosis, were identified on the upper limbs with extension to wrists, lower abdomen, groin, legs, buttocks, posterior thighs and knee folds.

Upon admittance, the patient had a BMI of 21.9 kg/m^2^, discolored hair and alopecia, brittle nails, hypotrophy of papillae lingual, tongue hyperemia and sacral and lower-limb edema. There was a symmetrical decrease in the sensitivity, motor and reflex functions of the lower limbs. She presented generalized erythematous desquamative plaque lesions with fine scales, interspersed with xerosis, without affecting the dorsum and palms of the hands (Figure 1).

Biochemical evaluation made it possible to rule out endocrine, renal or hepatic disorders that might interfere in zinc absorption or excretion. Analysis of stool fat by means of fat-soluble dye Sudam III was positive on two different occasions, thus characterizing an intestinal malabsorptive condition. Except for hypocalcemia (total Ca = 7.9 mg/dl; reference range (RR) = 8.4 to 10.5 mg/dl) and slight hypokalemia (K = 3.3 mmol/l; RR = 3.5 to 5.0 mmol/l), the other electrolytes (Na, P and Mg) were normal. Normocytic normochromic anemia (red blood cell = 9.8 g/dl) was detected, with iron serum levels close to the lower normal limit (iron = 40 mg/dl; RR = 35 to 150 mg/dl). Laboratory tests revealed protein undernutrition, with hypoproteinemia (total proteins = 5.2 g/dl; RR = 6.4 to 8.2 mg/dl), hypoalbuminemia (albumin = 2.3 g/dl; RR = 3.5 to 5.0 mg/dl), and reduced transferrin serum levels (61.8 mg/dl; RR = 195-313 mg/dl). A 34% decrease in body muscle mass was estimated with respect to normal values, as calculated from 24-hour urine creatinine levels. Neither folic acid (7.7 ng/dl; RR = 3 to 17 ng/ml) nor vitamin B_12_ (> 1,000 pg/ml; RR = 174-879 pg/ml) deficiencies were identified. Reduced copper (31 mg%, RR = 70 to 140 mg%) and ceruloplasmin (0.12 g/dl, RR = 0.2 to 0.55 g/dl) plasma levels were found, which characterized hypocupremia. Low zinc plasma levels were observed (34.4 mg%; RR = 50 to 120 mg%), as measured by atomic absorption spectroscopy conducted on Perkin-Elmer 290B equipment. 

Since the first day of hospital stay, the patient had been receiving an antihistaminic agent (dexchlorpheniramine), corticosteroids (hydrocortisone 1%) and an antifungal drug (ketoconazole 2%). Because of the patient’s low acceptance of an oral diet and her malabsorptive condition, a semi-hydrolyzed enteral diet via a nasoenteral tube was prescribed, in order to ensure adequate nutritional supply. The patient received thiamine and copper sulfate supplementation. Despite the improvements to her nutritional and neurological status, the cutaneous lesions persisted. 

On the 25^th^ day after hospital admission, the patient was re-evaluated by a dermatological team. Thereafter, a daily dose of 1,000 mg of zinc sulfate was administered through a nasoenteral tube because of absence of viable venous accesses and the patient’s refusal to swallow the zinc tablets. One week later, it was seen that there had been a remarkable recovery of the dermatological picture. The patient was discharged from the hospital two months after admission, for follow-up at the outpatient clinic with oral diet.

## DISCUSSION

Initially, the dermatological symptoms were attributed to protein and vitamin undernutrition, but there was no improvement in the lesions after vitamin supplementation and provision of adequate energy and protein supplies. The cutaneous lesion features, the low zinc plasma levels and the improvement in the dermatological status following zinc sulfate supplementation enabled a diagnosis of acquired zinc deficiency subsequent to bariatric surgery. 

It has been reported that energy restriction induces reduction in urinary zinc excretion^17^ and mobilization of this mineral from adipose tissue,^18^ which in turn results in increased zinc plasma levels. Decreased zinc concentration six months after Roux-en-Y gastric bypass (RYGBP), compared with preoperative values (69.8 ± 10.9 g/dl versus 93.2 ± 19.3 g/dl, respectively), has been documented.^19^ In one study, low zinc plasma levels were found in 68% of the patients undergoing RYGBP, two months after the operation, although 71% of the obese individuals had presented zinc deficiency prior to surgery, which was ascribed to low zinc intake.^17^ Regardless of the surgical technique (biliopancreatic diversion or duodenal switch), it has been reported that the incidence of zinc deficiency is 51%, one year after surgery, and remains at a similar level thereafter, with a 50% incidence four years after surgery.^7^ Some early studies showed hypozincemia in teenagers^20^ and adults,^8^ whereas another showed no significant difference with regard to zinc^21^ in patients after jejunoileal bypass. 

Even though there are many papers describing zinc deficiency after bariatric surgery, studies that document cutaneous manifestation of zinc deficiency after bariatric surgery are scarce (Table 1). We made a systematic search for indexed articles published on this topic and our patient is the second reported case with this condition. Kwashiorkor, Zn deficiency and an acrodermatitis enteropathica-like eruption were previously reported in one gastric bypass patient who failed to take nutrient supplements.^9^

**Table 1.** Search strategies performed on March 8, 2011, and results from Medline, Lilacs (Literatura Latino Americana e do Caribe em Ciências da Saúde), IBECS (Índice Bibliográfico Espanhol em Ciências da Saúde) and the Cochrane Library regarding the topic of zinc deficiency after bariatric surgery with or without acrodermatitis

Database	Search terms	Results	Relevant findings
PubMed	(zinc deficiency) AND (bariatric surgery) OR (zinc deficiency) AND (obesity)	9 articles 3 reviews	laboratory zinc deficiency was reported without acrodermatitis
	(zinc deficiency) AND (jejunoileal bypass)	3 articles	
	(zinc deficiency) AND (acrodermatitis) AND (bariatric surgery)	1 case report	acrodermatitis enteropathica-like eruption six months after undergoing a distal Roux-en-Y gastric bypass procedure
Lilacs	(deficiência de zinco) AND (cirurgia bariátrica) OR (deficiência de zinco) AND (obesidade)	0 articles	there were no articles about the subject
	(deficiência de zinco) AND (derivação jejuno-ileal) OR (derivação intestinal)	0 articles	
	(deficiência de zinco) AND (acrodermatite) AND (cirurgia bariátrica)	0 articles	
IBECS	(deficiencia de zinc) AND (cirugía bariátrica) OR (deficiencia de zinc) AND (obesidad	2 articles	laboratory zinc deficiency was reported without acrodermatitis
	(deficiencia de zinc) AND (derivación yeyunoileal)	0 articles	
	(deficiência de zinc) AND (cirurgía bariátrica) AND (acrodermatitis)	0 articles	
Cochrane Library	(zinc deficiency) AND (bariatric surgery) OR (zinc deficiency) AND (obesity)	0 articles	there were no articles about the subject
	(zinc deficiency) AND (jejunoileal bypass)	0 articles	
	(zinc deficiency) AND (acrodermatitis) AND (bariatric surgery)	0 articles	

In the present case, it is possible to dismiss the possibility that this case consisted only of inadequate zinc intake one week prior to hospital admission. Reduced food consumption and zinc intake below half the recommended values are among the causes of zinc deficiency after bariatric surgery.^22^

Jejunoileal bypass surgery is a procedure that was commonly used to treat morbid obesity in the past, but this technique is no longer performed. RYGBP and biliopancreatic diversion are now commonly performed, and these cause predictable selective micronutrient deficiencies that can be avoided by early supplementation.^23^ Moreover, the combination of jejunoileal bypass and vertical gastroplasty has been banned from bariatric surgery procedures in Brazil and worldwide. In view of the need to regulate morbid obesity surgery, the Brazilian Medical Council determined in 2010 that jejunoileal bypass and its variations performed solely in the small intestine (malabsorptive surgery) should be outlawed, because of the high incidence of late metabolic and nutritional complications.^24^

The surgical technique that had been used in the present case preserved the duodenum and proximal jejunum, which is where physiological zinc absorption occurs. However, jejunoileal bypass usually produces diarrhea, which may have contributed towards the zinc deficiency presented in this case. Zinc deficiency may have been secondary to hypotrophy of the enterocytes (a consequence of protein deficiency), regardless of the intestinal segment that was excluded during the surgical intervention. In the case of protein undernutrition, the modifications to intestinal structure and function range from a slight decrease in villus height to severe hypotrophy of the enterocytes,^25^ which may culminate in diminished absorptive capacity. In addition, it is likely that the loss of digestive secretion through the enterocutaneous fistula that appeared in the immediate postoperative period contributed towards zinc depletion. 

Zinc is a natural structural component of the cytosolic enzyme superoxide dismutase^26^ and of a class of metallothionein,^27^ which both display antioxidant activity. In the present case, the oxidative stress stemming from surgery and repeated infections may have resulted in an increased metabolic demand for zinc-dependent antioxidants, thereby contributing towards the reduction in body zinc levels. 

Given that this patient had multiple nutritional deficiencies, it is possible that the cutaneous lesions resulted from the sum of zinc depletion and lack of other nutrients, such as proteins, copper and B-complex vitamins like thiamine and niacin. The low copper levels might account for the neurological picture^28^ and may have contributed towards anemia,^29^ but this is not compatible with the dermatological signs presented by the patient. The hypothesis that zinc deficiency alone was responsible for the skin manifestations is corroborated by the fact that, although therapeutic measures involving vitamin supplementation had already been taken, complete regression of the lesions only took place after one week of zinc supplementation.

## CONCLUSION

Bariatric surgeons should follow the recommendations of the Professional Council in choosing surgical procedures, in order to avoid predictable complications. The multiprofessional team needs to be familiar with the surgical procedure performed and should obtain a detailed account of the patient’s medical history and perform exhaustive physical examination and biochemical tests to identify any covert nutritional deficiencies. The present case emphasizes the importance of taking zinc deficiency into consideration in the differential diagnosis of cutaneous lesions among patients undergoing bariatric surgery, in order to be able to start early treatment.
